# A Splice-Variant Imbalance of Reticulon-like Protein 16 (RTNLB16) Disrupts Growth and Decreases Sensitivity to ABA and Dark-Induced Senescence in *Arabidopsis*

**DOI:** 10.3390/plants15132022

**Published:** 2026-06-30

**Authors:** Tami Khazma, Dikla Levi, Hiba Waldman Ben-Asher, Tamir Shechtman, Gal Nisan, Gad Miller

**Affiliations:** The Mina and Everard Goodman Faculty of Life Sciences, Bar-Ilan University, Ramat-Gan 5290002, Israel; tbarktel105@gmail.com (T.K.); diklalevi12@gmail.com (D.L.); hiba.waldman-ba@biu.ac.il (H.W.B.-A.); tamirt500@gmail.com (T.S.); gal.nissan@biu.ac.il (G.N.)

**Keywords:** *Arabidopsis*, reticulon, endoplasmic reticulum, plasmodesmata, T-DNA, CaMV35S, abscisic acid, senescence, photoperiodism

## Abstract

Reticulon-like proteins shape the endoplasmic reticulum (ER) membrane network, yet the developmental and physiological roles of individual plant reticulon isoforms remain poorly understood. Here, we characterize an *Arabidopsis RTNLB16* T-DNA allele, *rtnlb16-1*, that exhibits severe photoperiod-dependent growth retardation and chlorosis. Molecular analysis revealed that *rtnlb16-1* is not a simple loss-of-function mutant: the T-DNA insertion deletes the 5′ region required for *RTNLB16* splice variant 7, while a CaMV35S enhancer associated with the insertion drives overexpression of the remaining splice variants. This misexpression is enhanced under long-day photoperiods and reduced under continuous low light, paralleling the severity of the mutant phenotype and its partial rescue. RTNLB16.5-GFP localized mainly to the tubular ER network and punctate cell-boundary structures consistent with plasmodesmata-associated ER. Neither overexpression of RTNLB16 isoforms 1–6 nor CRISPR-Cas9 disruption of major *RTNLB16* isoforms reproduced the *rtnlb16-1* phenotype, supporting a model in which altered splice-variant stoichiometry, rather than simple loss or gain of function, underlies the developmental defects. Transcriptome profiling showed that *rtnlb16-1* undergoes extensive photoperiod-dependent transcriptional reprogramming, including changes in defense, hormone-response, senescence, photosynthesis, and iron/redox-associated gene networks. Physiologically, *rtnlb16-1* displayed enhanced recovery from dark-induced senescence, while both *rtnlb16-1* and *rtnlb16-2* showed reduced sensitivity to exogenous abscisic acid during germination. Together, these findings suggest that balanced expression of *RTNLB16* splice variants is important for normal growth and for coordinating ER-associated stress, hormone, and senescence responses in *Arabidopsis*.

## 1. Introduction

Reticulon (RTN) family proteins are a class of highly conserved, endoplasmic reticulum (ER)-resident membrane-shaping proteins that participate in diverse cellular processes. The reticulon superfamily consists of reticulons (RTNs) in vertebrates and reticulon-like (RTNL) proteins in non-chordate eukaryotes, including the reticulon-like protein B (RTNLB) subfamily in plants [1].

The ER forms a dynamic membrane network of sheets and tubules, and reticulons help generate and stabilize the highly curved tubular regions through membrane insertion and oligomerization [2,3,4,5].

Structurally, reticulons are characterized by a conserved C-terminal reticulon homology domain (RHD) of approximately 200 amino acids. The RHD contains long hydrophobic, membrane-inserting segments separated by hydrophilic cytoplasmic loops, forming a hairpin-like topology that promotes membrane curvature and supports formation of the tubular ER network [6,7].

Ref. [6] Compared with the RHD, the N-terminal regions of reticulons are highly divergent, suggesting that these regions confer functional specificity by mediating interactions with distinct local binding partners [8].

Reticulon proteins participate in diverse eukaryotic cellular processes, including membrane organization, ER-to-Golgi trafficking, vesicle budding, membrane fusion, and apoptosis [5,6,7]. For example, mammalian RTN3 functions as an ER-phagy receptor during the selective turnover of ER tubules [9], whereas mammalian RTN4a/Nogo-A can act at the cell surface to regulate neurite growth and plasticity in the central nervous system [1,10].

In plants, *RTNLB* genes are substantially more numerous and diverse than in mammals or fungi, likely due to ancestral whole-genome duplication events [11,12]. Beyond standard ER localization, several plant RTNLB proteins localize to desmotubules, the narrow tubes of appressed ER that traverse plasmodesmata (PD), suggesting specialized roles in shaping PD structural architecture and modulating intercellular communication [13].

Many plant *RTNLB* genes have complex genomic structures with multiple introns, making them subject to alternative splicing. According to the *Arabidopsis* Information Resource (TAIR), most *RTNLB* loci produce two or more splice variants. Notably, *RTNLB11* and *RTNLB16* are the most splice-diverse members, each generating seven splice variants that may encode distinct protein isoforms. Despite this structural diversity, the specific physiological roles of individual RTNLB splice variants remain largely uncharacterized. Moreover, it is unknown whether RTNLB splice variants function independently, redundantly, or in defined stoichiometric relationships required for normal plant growth and stress responsiveness.

In this study, we characterize the *Arabidopsis thaliana* T-DNA mutant *rtnlb16-1*, which exhibits severe growth retardation and chlorosis that are strongly exacerbated by light–dark cycles and attenuated under continuous low-light conditions. Molecular analysis revealed that this allele disrupts the 5′ region of *RTNLB16* splice variant 7, while a T-DNA-associated CaMV35S enhancer drives photoperiod-dependent overexpression of the remaining splice variants. Genetic analyses showed that neither transgenic overexpression of isoforms 1–6 nor CRISPR-Cas9 disruption of major RTNLB16 isoforms is sufficient to reproduce the *rtnlb16-1* phenotype, supporting a model in which altered splice-variant stoichiometry underlies the mutant defects. Consistent with this model, transcriptome profiling uncovered photoperiod-dependent reprogramming of gene networks associated with stress, hormones, senescence, photosynthesis, and iron/redox response. Physiologically, *rtnlb16-1* exhibited reduced sensitivity to dark-induced senescence, whereas both *rtnlb16-1* and *rtnlb16-2* showed reduced sensitivity to exogenous ABA during germination. Together, these findings suggest that balanced expression of RTNLB16 splice variants is important for normal vegetative growth and for coordinating ER-associated stress and hormone responses in *Arabidopsis.*

## 2. Results

### 2.1. Salk_122275 Is a Photoperiod-Dependent RTNLB16 Allele Rather than an MSD1 Mutant

**Figure 1:**
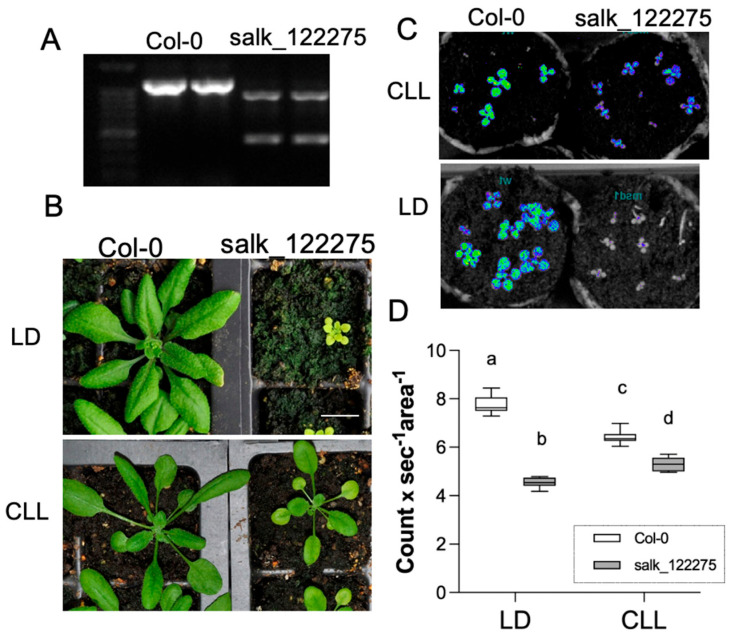
Characterization of the *rtnlb16-1* mutant phenotype under different light regimes. (**A**) PCR genotyping of Salk_122275 compared with wild-type Col-0, confirming recovery of homozygous mutant plants. (**B**) Representative four-week-old Col-0 and Salk_122275 plants grown under long-day conditions (LD; 16 h light/8 h dark) or continuous low light (CLL). Under LD, the mutant shows pronounced growth retardation and chlorosis, whereas growth under CLL partially alleviates both phenotypes. Scale bar = 1 cm. (**C**) Delayed-fluorescence images of whole rosettes grown under CLL or LD conditions, used as a non-destructive proxy for chlorophyll-associated photosynthetic signal. (**D**) Quantification of delayed fluorescence per unit area under each light condition. Data are shown as plotted; bars represent the mean ± SD of 8 biological replicates; different letters indicate statistically significant differences among groups (two-way ANOVA followed by Tukey’s multiple-comparisons test, *p* < 0.05).

The *Arabidopsis* Salk_122275 is annotated in the T-DNA express mapping tool [14] as an insertion within the first exon of the mitochondrial manganese superoxide dismutase 1 (MSD1) gene. Because MSD1 is a highly conserved and essential gene for normal development [15,16], we first examined whether the phenotype of this line was consistent with *MSD1* disruption. Homozygous Salk_122275 plants were recovered by PCR genotyping (Figure 1A), indicating that the line is viable and fertile. However, homozygous plants displayed a strong light-regime-dependent phenotype. Under long-day (LD; 16 h light/8 h dark; 80–100 µmol m^−2^ s^−1^) conditions, rosettes were small and chlorotic, whereas growth under continuous low light (CLL; 40 µmol m^−2^ s^−1^) partially restored rosette expansion and greenness (Figure 1B). Chlorophyll-associated delayed fluorescence imaging confirmed a strong reduction in the photosynthetic/chlorophyll signal under LD and partial recovery under CLL (Figure 1C,D).

**Figure 2:**
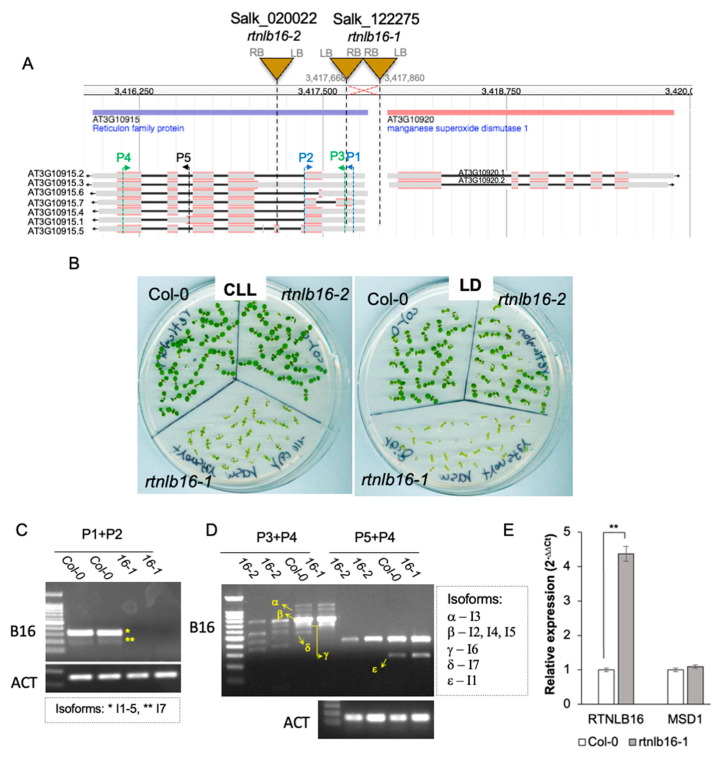
Identification of *RTNLB16* T-DNA alleles and splice-variant expression. (**A**) Gene model of the *RTNLB16* (AT3G10915) locus showing the seven annotated splice variants, the neighboring *MSD1* (AT3G10920) locus, primer positions (P1–P5), and the T-DNA insertion sites in Salk_122275/*rtnlb16-1* and Salk_020022/*rtnlb16-2*. Boxes and lines denote exons and introns, respectively, and transcript orientation is indicated in the gene model. In *rtnlb16-1*, the insertion/deletion affects the 5′ region required for *RTNLB16* isoform 7, whereas *rtnlb16-2* carries a T-DNA insertion within the *RTNLB16* intronic region. (**B**) Representative Col-0, *rtnlb16-1*, and *rtnlb16-2* seedlings grown on plates under CLL or LD conditions. The *rtnlb16-1* allele displays severe light-regime-dependent growth inhibition, whereas *rtnlb16-2* is near wild-type in appearance. (**C**,**D**) Semi-quantitative RT-PCR analysis of *RTNLB16* splice-variant expression using the primer combinations indicated in panel A. *ACTIN* was amplified as a cDNA/loading control. Greek letters annotate the PCR bands corresponding to the isoform groups amplified by each primer pair. (**E**) qRT-PCR analysis of total *RTNLB16* and *MSD1* transcript abundance in CLL-grown Col-0 and *rtnlb16-1* plants. Expression values were normalized to *ACTIN* and are shown relative to Col-0. Bars represent the mean ± SE of 3 biological replicates; ** *p* < 0.01.

To test whether MSD1 was affected, we measured MSD1 protein abundance and SOD activity. Immunoblot analysis with anti-MSD1 antibodies and native SOD activity assays showed comparable MSD1 protein levels and activity in Salk_122275 and Col-0 (Figure S1). Thus, the visible phenotype is unlikely to result from loss of MSD1 function. Restriction site extension PCR (RSE-PCR) was then used to map the T-DNA insertion sites. This analysis identified two insertions: one intergenic insertion on chromosome 4, matching the database annotation, and a second insertion on chromosome 3 within the compact genomic region containing *RTNLB16* (AT3G10915) and *MSD1* (AT3G10920) on opposite strands (Figure 2A and Figure S2). The chromosome 3 insertion consisted of a head-to-head T-DNA concatemer and was associated with a 191-bp genomic deletion that removed part of the promoter/5′ UTR region and the first 35 nucleotides of the predicted open reading frame of *RTNLB16* isoform 7 (Figure 2A and Figure S2). The chromosome 4 insertion was segregated away, and all subsequent analyses were performed with the line carrying the *RTNLB16*-associated insertion. We therefore refer to this allele as *rtnlb16-1*.

### 2.2. rtnlb16-1 Produces an RTNLB16 Splice-Variant Imbalance

RTNLB16 is predicted to produce seven splice variants. Semi-quantitative RT-PCR showed that isoform 7 was not detected in *rtnlb16-1*, whereas transcripts corresponding to isoforms 1–6 were retained and appeared elevated relative to Col-0 (Figure 2C,D). qRT-PCR confirmed increased total *RTNLB16* transcript abundance in *rtnlb16-1*, with approximately fourfold overexpression under CLL (Figure 2E). This pattern is consistent with activation of neighboring genomic sequences by CaMV35S promoter/enhancer elements present in Salk T-DNA insertions [17].

We also examined an independent T-DNA allele, Salk_020022/*rtnlb16-2*, which carries an insertion in the first intron of *RTNLB16* (Figure 2A and Figure S3). This allele disrupts or reduces the expression of several *RTNLB16* splice variants in a different combination pattern than *rtnlb16-1*. In contrast to *rtnlb16-1*, *rtnlb16-2* plants were visually similar to Col-0 under both CLL and LD conditions (Figure 2B and Figure S3). These results indicate that the severe *rtnlb16-1* phenotype is not explained by simple disruption of the *RTNLB16* locus. Instead, *rtnlb16-1* combines loss of isoform 7 with elevated expression of the remaining isoforms.

### 2.3. Growth Inhibition in rtnlb16-1 Is Driven by Light-to-Dark Transitions

**Figure 3:**
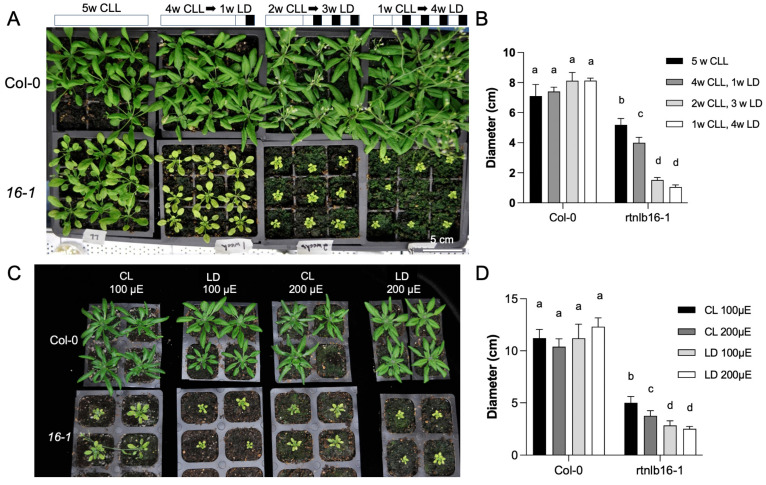
Photoperiod-dependent growth inhibition in *rtnlb16-1*. (**A**) Representative plants showing the effect of increasing exposure to LD conditions. Col-0 and *rtnlb16-1* plants were germinated and initially grown under CLL, then either maintained under CLL for five weeks or transferred to LD after four, two, or one week of CLL growth, resulting in one, three, or four weeks of LD exposure, respectively. All plants were photographed at five weeks of age. (**B**) Rosette diameter quantification for the plants shown in panel A. (**C**) Representative plants used to distinguish the effects of light regime from light intensity. Two-week-old CLL-grown Col-0 and *rtnlb16-1* plants were transferred for two weeks to continuous light (CL) or LD at either 100 or 200 µmol m^−2^ s^−1^. (**D**) Rosette diameter quantification for the plants shown in panel C. Bars represent the mean ± SD with 8 replicates. Different letters indicate statistically significant differences among groups (two-way ANOVA followed by Tukey’s multiple-comparisons test, *p* < 0.01).

Because CLL partially rescued *rtnlb16-1*, we next tested how LD exposure affects growth. Plants were germinated and initially grown under CLL, then either maintained under CLL or transferred to LD for increasing durations before imaging at five weeks of age. *rtnlb16-1* plants grown continuously under CLL were smaller than Col-0 but remained relatively green. In contrast, increasing the duration of LD exposure progressively reduced mutant rosette size and enhanced chlorosis (Figure 3A,B). After one week under LD, *rtnlb16-1* rosettes were approximately half the size of Col-0. After three to four weeks under LD, mutant rosettes were reduced to approximately 20–25% of the corresponding wild-type size. Reciprocal transfer experiments further showed that the phenotype worsened after transfer from CLL to LD and partially recovered after return from LD to CLL (Figure S4). To distinguish the effect of photoperiod from total light intensity, two-week-old CLL-grown plants were transferred to either continuous light (CL) or LD at 100 or 200 µmol m^−2^ s^−1^. Col-0 growth was comparatively stable across these conditions. By contrast, *rtnlb16-1* plants were consistently more impaired under LD than under CL at the same light intensity (Figure 3C,D). At 100 µmol m^−2^ s^−1^, LD-grown *rtnlb16-1* plants reached approximately 25% of the wild-type rosette diameter, compared with approximately 45% under CL. At 200 µmol m^−2^ s^−1^, LD-grown mutants reached approximately 20% of wild-type size, compared with approximately 36% under CL. Similar photoperiod-dependent differences were observed in flowering plants and inflorescence development (Figure S5). These data indicate that the day–night cycle, rather than light intensity alone, is the primary environmental factor that enhances the *rtnlb16-1* growth defect.

### 2.4. RTNLB16.5 Localizes to the ER Network and Punctate Structures at Cell Boundaries

**Figure 4:**
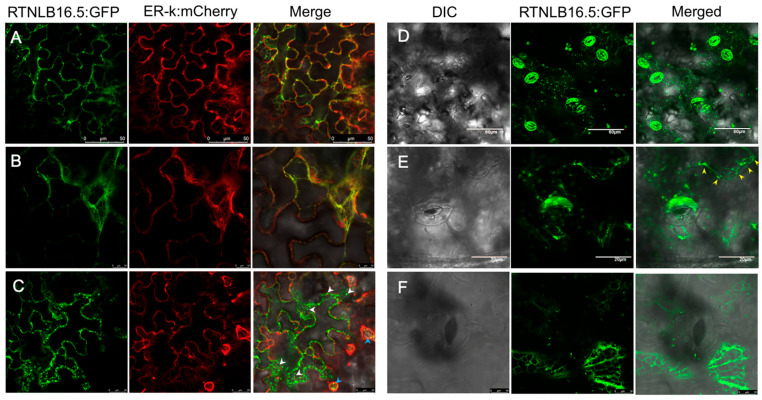
Subcellular localization of RTNLB16.5-GFP in plant cells. (**A**–**C**) Confocal images of *Nicotiana benthamiana* leaf epidermal cells transiently co-expressing RTNLB16.5-GFP and the ER marker ER-rk:mCherry. GFP, mCherry, and merged channels are shown. RTNLB16.5-GFP labels the cortical ER network and cell periphery and partially overlaps with ER-rk:mCherry; punctate RTNLB16.5-GFP accumulations are visible in some regions. White arrowheads in C point to cytosolic GFP puncta suspected as golgi bodies. (**D**–**F**) Confocal images from *Arabidopsis* plants expressing RTNLB16.5-GFP. DIC, GFP, and merged channels are shown. RTNLB16.5-GFP decorates ER-like networks, shows a prominent signal in guard cells, and accumulates in punctate structures at cell boundaries or pit fields, consistent with plasmodesmata-associated localization. Scale bars are shown in the panels.

To examine RTNLB16 subcellular localization, RTNLB16 isoform 5 was fused to GFP and expressed in plant cells. In transiently transformed *Nicotiana benthamiana* leaf epidermal cells, RTNLB16.5-GFP partially overlapped with the ER marker ER-rk:mCherry [18] and labeled the cortical ER network (Figure 4A–C and Figure S6). In addition to the ER-associated signal, RTNLB16.5-GFP accumulated in discrete punctate structures, including puncta near cell boundaries. In stable *Arabidopsis* lines, RTNLB16.5-GFP decorated ER-like networks, was prominent in guard cells, and was detected in structures consistent with pit fields or plasmodesmata-associated domains (Figure 4D–F). Together, these observations support an ER-associated localization for RTNLB16.5 and suggest that a fraction of the protein may accumulate at specialized ER–plasma membrane or plasmodesmatal sites.

### 2.5. Photoperiod-Sensitive CaMV35S Activity Correlates with RTNLB16 Overexpression and with Phenotype Severity

**Figure 5:**
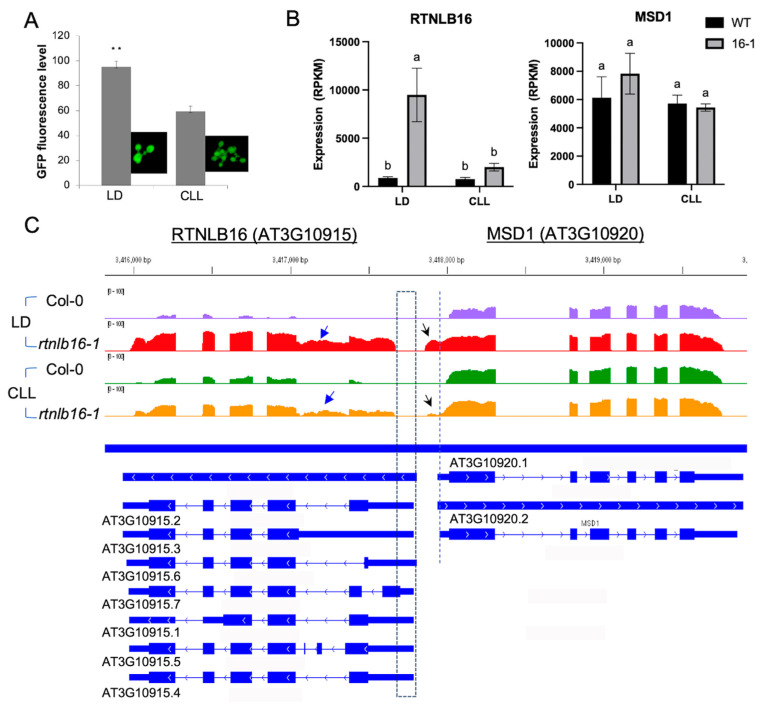
Photoperiodic regulation of the CaMV35S promoter and *RTNLB16* transcript levels. (**A**) Quantification of GFP fluorescence in CaMV35S:GFP reporter seedlings grown under LD or CLL, with representative GFP images shown in the graph. Higher fluorescence under LD indicates stronger CaMV35S-driven expression under photoperiodic conditions. (**B**) RNA-seq transcript abundance, expressed as RPKM, for *RTNLB1*6 and *MSD1* in Col-0 and *rtnlb16-1* plants grown under LD or CLL. RTNLB16 expression is strongly elevated in *rtnlb16-1*, particularly under LD, whereas *MSD1* transcript levels remain comparable between genotypes. (**C**) Integrated Genomics Viewer (IGV) read-coverage tracks across the *RTNLB16*–*MSD1* genomic region. Tracks show Col-0 and *rtnlb16-1* read coverage under LD and CLL, with the *RTNLB16* splice variant and *MSD1* gene models shown below. The dashed box marks the region absent in *rtnlb16-1* that overlaps the 5′ region of *RTNLB16* isoform 7. Arrows indicate regions of altered read coverage in the mutant. In panel (**A**), values are shown as the mean ± SD of 6 replicates, ** *p* < 0.01. In panel (**B**), values are shown as mean ± SE of 3 replicates; different letters indicate statistically significant differences among groups (two-way ANOVA followed by Tukey’s multiple-comparisons test, *p* < 0.01).

The combined absence of isoform 7 and elevated expression of isoforms 1–6 suggested that a transcriptional imbalance might underlie the *rtnlb16-1* phenotype. Because CaMV35S promoter activity can vary with light regime and photoperiod [19,20], we measured GFP fluorescence in a CaMV35S:GFP reporter line grown under CLL or LD. GFP fluorescence was higher under LD than under CLL (Figure 5A), indicating that CaMV35S-driven expression is enhanced under the photoperiodic condition that also produces the strongest *rtnlb16-1* phenotype.

RNA-seq analysis supported this relationship. *RTNLB16* transcript abundance increased approximately 2.6-fold in *rtnlb16-1* under CLL and approximately 11-fold under LD relative to Col-0 (Figure 5B). Integrative Genomics Viewer (IGV) read-coverage analysis showed elevated coverage across most *RTNLB16* exons in *rtnlb16-1*, except in the deleted region near the 5′ end of isoform 7 (Figure 5C). Although reads were also detected across the intergenic/promoter region adjacent to *MSD1*, *MSD1* transcript abundance remained similar between Col-0 and *rtnlb16-1* (Figure 5B,C). These results link the severity of the *rtnlb16-1* phenotype to photoperiod-enhanced *RTNLB16* overexpression and support the conclusion that *MSD1* expression is not substantially altered.

### 2.6. Neither RTNLB16 Overexpression nor RTNLB16 Knockout Alone Reproduces the rtnlb16-1 Phenotype

To test whether overexpression of *RTNLB16* isoforms 1–6 is sufficient to inhibit growth, we generated transgenic *Arabidopsis* lines expressing the *RTNLB16* genomic region containing isoforms 1–6 under either the constitutive CaMV35S promoter or a β-estradiol-inducible promoter. Although these lines accumulated higher *RTNLB16* transcript levels, they did not exhibit the severe growth retardation or chlorosis characteristic of *rtnlb16-1* under either CLL or LD (Figure S7).

We also examined whether loss of *RTNLB16* isoforms could explain the phenotype. A CRISPR-Cas9 allele, *rtnlb16-cr*, was isolated from a multiplex *RTNLB* gene-family targeting library. The guide RNA targeted a coding region shared by isoforms 1–5 and isoform 7 (Figure S8A). Sequencing revealed a single thymidine insertion one nucleotide upstream of the PAM, predicted to cause a frameshift and premature stop codon in the targeted isoforms while leaving isoform 6 unaffected (Figure S8B). Despite disruption of major *RTNLB16* isoforms, *rtnlb16-cr* plants were visually indistinguishable from Col-0 under the tested CLL and LD conditions (Figure S8C). Thus, neither overexpression of isoforms 1–6 nor loss of several major *RTNLB16* isoforms is sufficient to reproduce *rtnlb16-1*. The data support a model in which the specific combination of isoform 7 loss and elevated expression of the remaining isoforms produces the mutant phenotype.

### 2.7. RTNLB16 Imbalance Is Associated with Photoperiod-Enhanced Reprogramming of the Transcriptome

**Figure 6:**
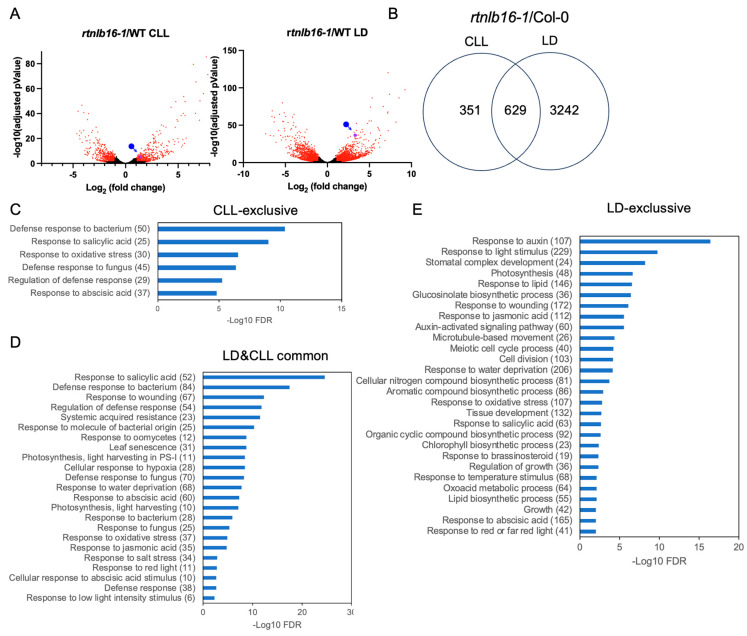
Photoperiod-dependent transcriptome reprogramming in *rtnlb16-1*. (**A**) Volcano plots comparing transcript abundance in *rtnlb16-1* relative to Col-0 under CLL and LD conditions. The X-axis shows log_2_ fold change, and the Y-axis shows −log_10_ statistical significance. Significantly differentially expressed genes (DEGs) are shown in red. *RTNLB16* is indicated by purple dots with blue circles and arrows pointing to their locations. DEGs were defined using the thresholds described in Section 4: absolute log_2_ fold change > 1 and *p* < 0.01. (**B**) Venn diagram summarizing overlap between DEGs detected under CLL and LD conditions. (**C**–**E**) Gene Ontology biological-process enrichment for the 351 CLL-exclusive DEGs (**C**), 629 DEGs common to CLL and LD (**D**), and the 3242 LD-exclusive DEGs (**E**). Bars indicate −log_10_ false-discovery rate (FDR), and numbers in parentheses indicate the number of genes assigned to each enriched term.

To identify molecular pathways associated with *RTNLB16* imbalance, we performed RNA-seq on four-week-old Col-0 and *rtnlb16-1* plants grown under CLL or LD. The number of differentially expressed genes (DEGs) was substantially higher under LD than under CLL. In total, 980 DEGs were detected in *rtnlb16-1* under CLL and 3871 under LD, with 629 DEGs shared between the two light regimes (Figure 6A,B). This larger LD-responsive transcriptional shift is consistent with the stronger *RTNLB16* overexpression and more severe phenotype observed under LD.

GO enrichment analysis revealed both shared and condition-specific responses. CLL-exclusive DEGs were enriched for defense-related processes, including salicylic acid (SA)- and abscisic acid (ABA)-associated responses (Figure 6C; Table S2). The 629 DEGs common to CLL and LD were enriched for SA-related defense responses, biotic stress pathways, leaf senescence, photosynthesis, and jasmonic acid (JA) signaling (Figure 6D; Table S3). LD-exclusive DEGs encompassed a broader set of processes, including auxin and light responses, photosynthesis, cell division, and wounding (Figure 6E; Table S4). These results indicate that *RTNLB16* imbalance affects stress-, hormone-, and development-associated transcriptional programs, with a particularly strong effect under photoperiodic conditions.

**Figure 7:**
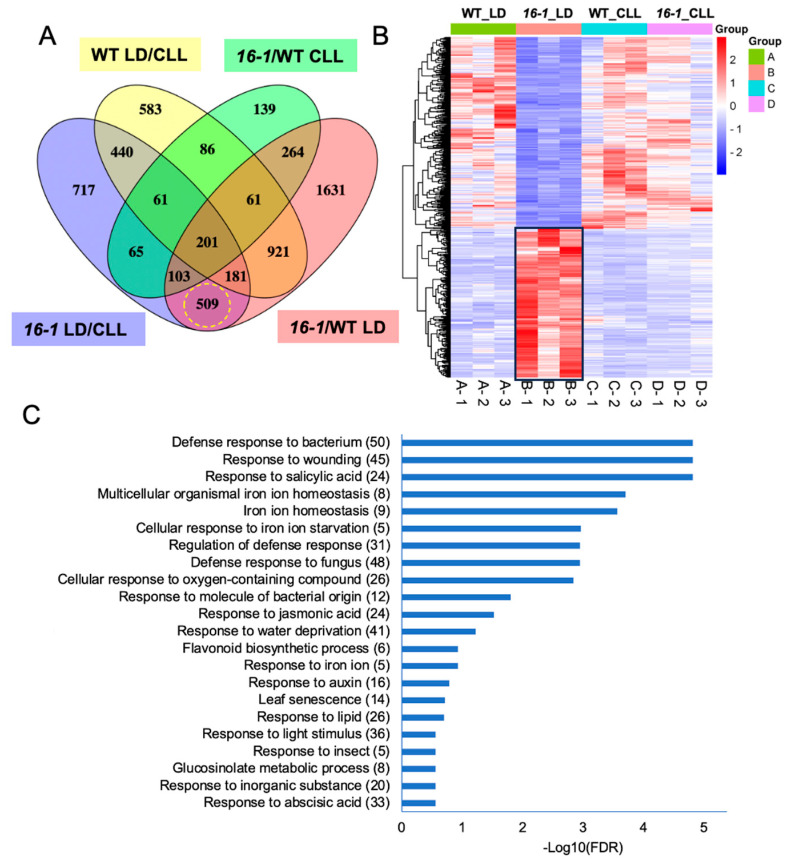
Analysis of a 509-gene subset associated with the LD-dependent *rtnlb16-1* phenotype. (**A**) Four-way Venn diagram comparing DEG sets from Col-0 LD versus Col-0 CLL, *rtnlb16-1* versus Col-0 under CLL, *rtnlb16-1* LD versus *rtnlb16-1* CLL, and *rtnlb16-1* versus Col-0 under LD. The dashed circle marks the 509-gene subset selected for downstream analysis because these genes are misregulated in LD-grown *rtnlb16-1* and return toward wild-type expression under CLL. (**B**) Heatmap of normalized expression values for the 509 genes across all biological replicates of WT_LD, *rtnlb16-1*_LD, WT_CLL, and *rtnlb16-1*_CLL. Rows represent genes and columns represent biological replicates; the boxed region highlights the altered expression pattern in LD-grown *rtnlb16-1*. (**C**) GO biological-process enrichment analysis of the 509-gene subset. Bars indicate −log_10_ FDR, and numbers in parentheses indicate the number of genes assigned to each term. Enriched processes include defense responses, salicylic acid and jasmonic acid responses, iron-ion homeostasis, senescence, and ABA-related responses.

To focus on genes most closely associated with the reversible LD-dependent phenotype, we identified a subset of 509 genes that were misregulated in LD-grown *rtnlb16-1* but returned toward wild-type expression levels under CLL (Figure 7A,B). GO enrichment analysis of this subset highlighted defense responses, SA and JA signaling, auxin-related development, leaf senescence, ABA responses, and iron homeostasis-related terms (Figure 7C; Table S5). Among the genes contributing to the iron/redox-associated enrichment were FERRITIN 1 (FER1; AT5G01600), FERRITIN 4 (FER4; AT2G40300), the Fe-S cluster-associated gene NEET (AT5G51720), and several bHLH transcription factors (Table S6), suggesting that iron/redox-associated transcriptional programs are altered in LD-grown *rtnlb16-1*.

### 2.8. rtnlb16-1 Shows Enhanced Recovery from Dark-Induced Senescence, Whereas Both rtnlb16 Alleles Show Reduced Sensitivity to ABA

**Figure 8:**
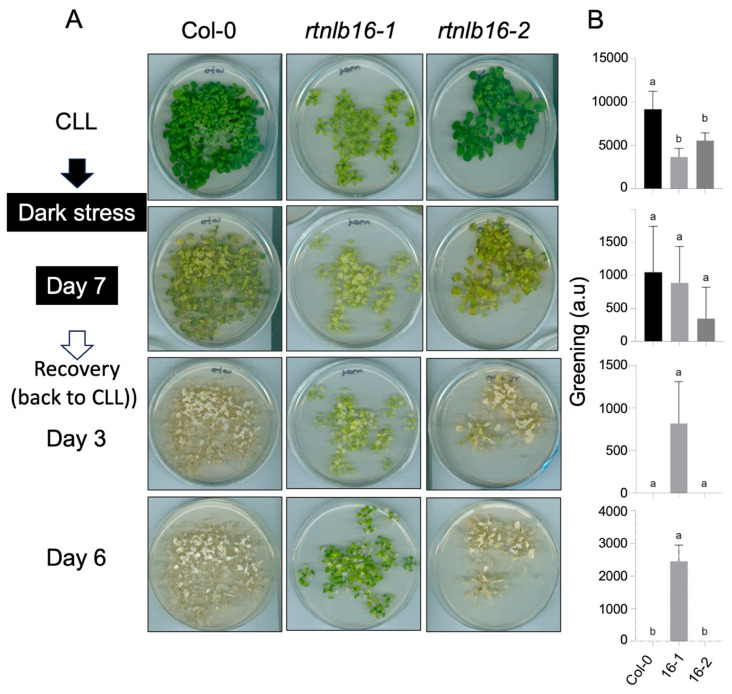
Dark-induced senescence and recovery in *rtnlb16* mutants. (**A**) Representative plates of Col-0, *rtnlb16-1*, and *rtnlb16-2* seedlings grown for 18 days under CLL, subjected to seven days of complete darkness, and then returned to CLL for recovery. Images show plants before dark stress, after seven days of darkness, and after three and six days of recovery. (**B**) Quantification of green pigmentation from the corresponding images using HSL-based image analysis. Greening values are reported in arbitrary units and reflect the abundance and intensity of green pixels. The *rtnlb16-1* mutant retains or re-establishes green pigmentation during recovery, whereas Col-0 and *rtnlb16-2* remain largely bleached. Bars represent the mean ± SD of 8 replicates; different letters indicate statistically significant differences among genotypes within each time point (one-way ANOVA followed by Tukey’s multiple-comparisons test, *p* < 0.05).

To test whether *RTNLB16* imbalance affects senescence-associated stress responses, 18-day-old seedlings grown on MS agar under CLL were transferred to complete darkness for seven days, then returned to CLL for recovery. After dark treatment and re-illumination, Col-0 and *rtnlb16-2* seedlings exhibited extensive bleaching and failed to recover. In contrast, *rtnlb16-1* seedlings retained or re-established green pigmentation during recovery and regained greenness within six days (Figure 8A). Image-based quantification confirmed that *rtnlb16-1* had significantly higher green pigmentation than Col-0 or *rtnlb16-2* during recovery (Figure 8B).

**Figure 9:**
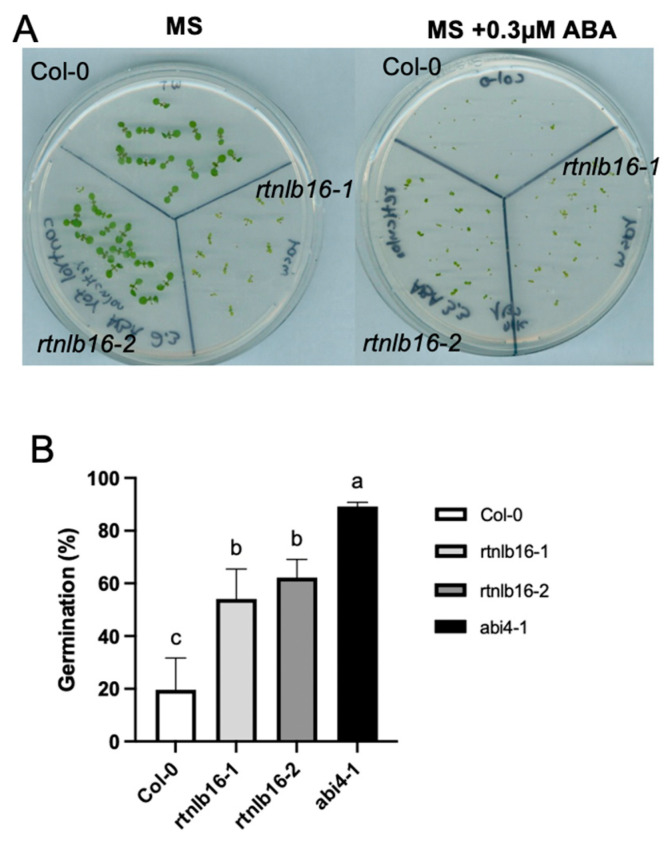
Reduced ABA sensitivity during germination in *rtnlb16* mutants. (**A**) Representative germination phenotypes of Col-0, *rtnlb16-1*, and *rtnlb16-2* seeds on MS medium with or without 0.3 µM ABA, photographed seven days after imbibition. (**B**) Germination percentage on MS medium containing 0.3 µM ABA five days after imbibition. The ABA-insensitive *abi4-1* mutant served as a positive control. Germination was scored by radicle emergence. Error bars represent mean ± SD of 4 replicates of 50 seeds; different letters indicate statistically significant differences among genotypes (one-way ANOVA followed by Tukey’s multiple-comparisons test, *p* < 0.05).

We next tested ABA sensitivity during germination by sowing seeds on MS medium containing 0.3 µM ABA and scoring radicle emergence five days after imbibition. Under these conditions, approximately 20% of Col-0 seeds germinated, whereas *rtnlb16-1* and *rtnlb16-2* reached approximately 60% germination (Figure 9). The ABA-insensitive *abi4-1* mutant showed the strongest response, with approximately 90% germination. These results indicate reduced ABA sensitivity in both *rtnlb16* alleles during germination. Together, these findings suggest that RTNLB16 contributes to stress-related processes affecting dark-induced senescence recovery and ABA sensitivity during germination.

## 3. Discussion

### 3.1. RTNLB16 Splice-Variant Imbalance Defines a Dosage-Sensitive Allele

Our main conclusion is that *rtnlb16-1* is not a conventional *RTNLB16* loss-of-function mutant. Instead, it is a splice-variant dosage allele in which the T-DNA insertion removes the 5′ regulatory and initiating region required for splice variant 7, while T-DNA-associated CaMV35S regulatory sequences drive elevated accumulation of the remaining *RTNLB16* splice variants. This interpretation is supported by isoform-specific RT-PCR data, RNA-seq coverage across the *RTNLB16* locus, and the strong photoperiod dependence of RTNLB16 transcript accumulation in the mutant. The behavior of the 35S:GFP reporter and previous reports that CaMV35S activity is influenced by photoperiod and darkness further support the idea that day–night cycles amplify *RTNLB16* misexpression in *rtnlb16-1* [17,19,20].

Overexpression of the genomic region encoding isoforms 1–6 in a wild-type background did not reproduce the severe growth phenotype, and CRISPR-Cas9 disruption of the major isoforms, including isoform 7, did not cause visible growth retardation under the conditions tested. These observations argue that neither *RTNLB16* overexpression nor *RTNLB16* loss alone is sufficient to explain *rtnlb16-1*. Together, the data support a dosage-imbalance model in which hyperaccumulation of isoforms 1–6 is deleterious only when isoform 7 is absent. A definitive test of this model would be complementation of *rtnlb16-1* with isoform 7, or reconstruction of the combined genotype by overexpressing isoforms 1–6 in an isoform-7-null background.

This interpretation is consistent with the general behavior of reticulon-family proteins. Reticulons shape the highly curved tubular ER by inserting long hydrophobic segments into membranes and assembling into relatively immobile oligomeric scaffolds [2,3,5]. Because oligomeric assembly is central to reticulon function, altered ratios among closely related isoforms could plausibly change the composition, geometry, or stability of ER-shaping complexes. Previous work has shown that reticulon overexpression can substantially remodel ER organization and restrict luminal diffusion, even when bulk secretion remains intact [21]. Thus, the *rtnlb16-1* phenotype may reflect an abnormal RTNLB16-containing membrane scaffold rather than a simple absence of RTNLB16 activity.

### 3.2. A Cellular Framework for RTNLB16 Function

The subcellular localization data provide a plausible structural context for the physiological defects. RTNLB16.5-GFP decorated the tubular ER network and was enriched at structures consistent with plasmodesmata, consistent with previous evidence that plant RTNLB proteins can localize to desmotubules and influence plasmodesmatal architecture [13]. The transiently expressed RTNLB16.5-GFP signal also included punctate structures that may correspond to specialized endomembrane subdomains, although additional co-localization with Golgi or other organelle markers would be needed to definitively assign these structures. These observations suggest that RTNLB16 may act at sites where ER membrane curvature, ER–plasma membrane contact, and intercellular communication intersect.

A disturbance at these sites could have broad downstream consequences. Plasmodesmata and the ER-desmotubule system facilitate the movement of developmental and stress-related signals between cells, while the ER supports secretory trafficking, protein quality control, lipid metabolism, and stress signaling. Therefore, the extensive transcriptomic reprogramming observed in *rtnlb16-1* is likely a secondary consequence of altered membrane organization rather than a direct transcriptional function of *RTNLB16*. The enrichment of gene sets related to defense, salicylic acid, jasmonic acid, ABA, auxin, senescence, and photosynthesis is consistent with a broad stress-acclimation state triggered by disruption of endomembrane or plasmodesmatal function.

### 3.3. Photoperiod-Dependent Transcriptome Reprogramming and Stress-Response Pathways

The light-regime experiments indicate that the severe *rtnlb16-1* phenotype is driven primarily by day–night transitions rather than by total light dose. Continuous low light substantially mitigated growth inhibition and chlorosis, whereas LD photoperiods increased *RTNLB16* transcript accumulation and exacerbated the phenotype. This pattern was also reflected at the transcriptome level, with many more DEGs detected under LD than under CLL. The 509-gene subset that returned toward wild-type expression under CLL therefore provides a useful candidate set of transcriptional changes associated with the reversible, photoperiod-sensitive component of the phenotype.

This recovered gene set was enriched for several stress- and development-related processes, including defense responses, SA and JA signaling, auxin-related development, ABA responses, senescence, photosynthesis-related processes, and iron-homeostasis-associated terms. These categories suggest that *RTNLB16* splice-variant imbalance affects multiple regulatory networks rather than a single downstream pathway. Among these responses, the induction of *FERRITIN 1*, *FERRITIN 4*, *NEET*, and several iron-associated *bHLH* transcription factors is consistent with altered regulation of iron/redox-associated gene expression in LD-grown *rtnlb16-1*. Ferritins and NEET-family proteins are linked to iron storage, Fe-S cluster biology, and redox-associated stress responses [22,23,24] and their altered expression may reflect a broader chloroplast or metabolic stress state associated with the chlorotic phenotype.

A working model is that LD conditions amplify the consequences of *RTNLB16* splice-variant imbalance through two non-exclusive mechanisms: increased CaMV35S-driven activation of *RTNLB16* isoforms 1–6 and repeated daily light–dark transitions. Together, these factors may perturb ER-associated cellular homeostasis and activate transcriptional programs associated with stress, hormones, senescence, photosynthesis, and iron/redox. Under CLL, reduced *RTNLB16* overexpression and the absence of abrupt light–dark transitions may lessen this transcriptional stress signature, thereby partially restoring growth and pigmentation.

### 3.4. ABA Sensitivity and Dark-Induced Senescence

The ABA and dark-induced senescence phenotypes provide additional evidence that RTNLB16 affects stress-related physiological responses. *rtnlb16-1* showed enhanced recovery after prolonged darkness, whereas Col-0 and *rtnlb16-2* exhibited extensive bleaching and poor recovery under the same conditions. In contrast, both *rtnlb16-1* and *rtnlb16-2* showed reduced sensitivity to exogenous ABA during seed germination. Thus, the ABA germination phenotype alone is insufficient to explain the *rtnlb16-1*-specific recovery from dark-induced senescence. Instead, reduced ABA sensitivity may reflect a broader role for RTNLB16 in ABA responsiveness, while the enhanced recovery of *rtnlb16-1* after dark treatment may depend on the specific splice-variant imbalance and the associated stress-response state of this allele.

This distinction is important because dark-induced senescence is regulated by multiple converging pathways, including ABA signaling, chloroplast retrograde signaling, sugar status, ROS-associated signaling, and senescence-associated transcriptional programs [25,26,27]. The ER may participate in this network because stress-activated ABA release can occur via beta-glucosidase-mediated hydrolysis of ABA-glucose ester in ER-associated compartments [28]. Given RTNLB16’s ER-associated localization, altered ER organization could plausibly influence one or more of these processes. However, resolving these possibilities will require direct measurements of endogenous ABA levels, expression of ABA metabolic and senescence-marker genes, chlorophyll catabolic gene activity, and plasmodesmatal permeability during dark treatment and recovery.

## 4. Materials and Methods

### 4.1. Plant Materials, Growth Conditions, and Treatments

All *Arabidopsis* lines used in this study were in the Columbia-0 (Col-0) background. The RTNLB16 T-DNA insertion lines Salk_122275 (*rtnlb16-1*) and Salk_020022 (*rtnlb16-2*) were obtained from the *Arabidopsis* Biological Resource Center (ABRC). Homozygous plants were identified by PCR genotyping with gene-specific and T-DNA border primers, following the SIGnAL/SAIL genotyping recommendations [14]. Primer sequences are listed in Table S1.

Plants were grown in Percival AR-66 growth chambers (Percival Scientific, Perry, IA, USA) or in temperature-controlled growth rooms at 23 °C and 70% relative humidity. Unless otherwise indicated, plants were grown under long-day conditions (LD; 16 h light/8 h dark; 80–100 µmol m^−2^ s^−1^) or continuous low light (CLL; 40 µmol m^−2^ s^−1^). Because *rtnlb16-1* is highly sensitive to photoperiod, plants used in transfer experiments were germinated and initially grown under CLL before exposure to LD or continuous light (CL) treatments.

For photoperiod-transfer assays, plants were germinated under CLL and transferred to LD for the indicated durations before phenotyping at the same age. For light-intensity experiments, two-week-old plants grown under CLL were transferred to LD or CL at 100 or 200 µmol m^−2^ s^−1^. Rosette diameter was measured from digital images along the rosette’s widest axis.

### 4.2. Mapping of T-DNA Insertion Sites

T-DNA insertion sites in Salk_122275 were mapped by restriction site extension PCR (RSE-PCR) as previously described [29]. PCR products were sequenced and aligned to the TAIR10 *Arabidopsis* genome to identify flanking genomic sequences. The *RTNLB16*-associated insertion and the 191-bp deletion were confirmed by PCR and sequencing using primers listed in Table S1. The unlinked chromosome 4 insertion was segregated away before subsequent phenotypic and molecular analyses.

### 4.3. ABA Germination Assay

Freshly harvested seeds were surface-sterilized and sown on half-strength MS agar medium, with or without 0.3 µM abscisic acid (ABA). Plates were incubated under CLL conditions. Germination was scored five days after imbibition based on radicle emergence. The ABA-insensitive *abi4-1* mutant served as a positive control.

### 4.4. Dark-Induced Senescence and Image-Based Quantification of Green Pigmentation

For dark-induced senescence assays, 18-day-old seedlings grown on MS agar plates under CLL were wrapped in aluminum foil and incubated in complete darkness in the growth chamber for seven days. Plates were then returned to CLL, and recovery was imaged at the indicated time points.

Green pigmentation was quantified from digital images using MATLAB (vR2025b). Plants were segmented from the background, and five non-overlapping circular regions of interest of similar size were selected per sample. Pixels in each region were converted from RGB to HSL color space. Green pixels were defined by hue values between 80 and 125 degrees and weighted by inverse lightness [green mask × (1 − lightness)] to reduce the contribution of pale or overexposed pixels. Pixel scores below 0.11 were set to zero, and the final greenness value for each sample was calculated from the five regions of interest.

### 4.5. RNA Extraction, RT-PCR, and qRT-PCR

Total RNA was extracted from fresh leaf tissue using TRIzol reagent (Life Technologies, Carlsbad, CA, USA) following the manufacturer’s instructions. cDNA synthesis, semi-quantitative RT-PCR, and quantitative real-time PCR (qRT-PCR) were performed as previously described [30]. Expression values were normalized to *ACTIN2* or to the internal control specified for the relevant experiment. Primer names and sequences are listed in Table S1.

### 4.6. SOD Activity Assays and Immunoblotting

Leaf protein extracts were prepared from 250 mg of tissue collected from three-week-old CLL-grown plants. For native SOD activity assays, 30 µg of total protein was resolved on a 10% non-denaturing polyacrylamide gel, and SOD activity was stained as described by [31]. Where indicated, gels were treated with 8 mM H_2_O_2_ to inhibit H_2_O_2_-sensitive SOD isoforms and enhance detection of H_2_O_2_-resistant MSD1 activity. Immunoblot analysis was performed as previously described [32] using a polyclonal anti-MSD1 antibody (Agrisera, Sweden; Product no. AS09 524) at a 1:3000 dilution. Coomassie-stained gels served as loading controls.

### 4.7. Cloning and Generation of Transgenic Plants

For subcellular localization, the coding sequence of RTNLB16 splice variant 5 was PCR-amplified and cloned into the Gateway pDONR221 entry vector, then recombined into the pH7FWG2 destination vector [33] to generate a C-terminal GFP fusion under the CaMV35S promoter. For overexpression analyses, the RTNLB16 genomic fragment encompassing the coding regions of isoforms 1–6 was cloned into pH7FWG2 and into the β-estradiol-inducible pMDC7 vector [34]. PCR amplification was performed using Herculase II Fusion DNA Polymerase (Agilent Technologies, Santa Clara, CA, USA ) with primers listed in Table S1.

The *rtnlb16-cr* CRISPR-Cas9 allele was isolated from a multiplex CRISPR library targeting the *Arabidopsis* RTNLB gene family, generated using the strategy described in [35]. The mutation was confirmed by PCR amplification and Sanger sequencing of the target region.

### 4.8. Transient Expression in N. benthamiana

Agrobacterium-mediated transient expression in *N benthamiana* leaves was performed as described [36]. For co-expression experiments, Agrobacterium cultures carrying RTNLB16.5-GFP and ER-rk:mCherry were mixed at a 1:1 ratio before infiltration.

### 4.9. Fluorescence Microscopy and Imaging

Confocal fluorescence imaging of infiltrated *N. benthamiana* leaf epidermal cells and transgenic *Arabidopsis* tissues was performed using an Olympus FV1000 confocal microscope. GFP imaging of CaMV35S:GFP *Arabidopsis* seedlings was performed using the CRI Maestro II live-imaging system (PerkinElmer, Waltham, MA, USA). Image acquisition settings were kept consistent within each experiment.

### 4.10. Delayed Fluorescence Imaging

Chlorophyll-associated delayed fluorescence was measured from entire rosettes of 14-day-old plants using a NightSHADE LB985 imager (Berthold Technologies, Bad Wildbad, Germany), as described by [37]. Delayed fluorescence signal was quantified per rosette area as indicated in the relevant figure.

### 4.11. RNA-seq and Bioinformatic Analyses

RNA-seq libraries were prepared as previously described [38]. Twelve samples, representing three biological replicates for each genotype and light condition, were sequenced on a single lane of an Illumina HiSeq 2500 sequencer (Illumina, Inc., San Diego, CA, USA) in high-output mode, yielding approximately 30–50 million reads per sample. Reads were aligned to the *Arabidopsis* reference genome (TAIR10/Araport11, release 06/2016) using STAR version 2.5.0a [39], resulting in 89.5–95.5% uniquely mapped reads, corresponding to 27.5–47.3 million uniquely mapped reads per sample [39]. Gene-level count matrices were generated with HTSeq version 0.6.1p1 [40], and differential expression was analyzed with DESeq2 [41]. Only reads that could be unambiguously assigned to a single genomic feature were included in the count matrix. No additional gene-level filtering was applied prior to differential expression analysis.

Differential expression was assessed for *rtnlb16-1* versus Col-0 within each light regime and for LD versus CLL within each genotype, using three biological replicates per group. Differentially expressed genes (DEGs) were defined as genes with an absolute log_2_ fold change > 1 and a *p* value < 0.01. Functional enrichment analysis was performed using DAVID Bioinformatics Resources 6.8 [42]. RNA-seq read coverage was visualized using IGV [43]. The RNA-seq data have been deposited in GEO under accession number GSE242257 and will be available on 1 September 2026.

### 4.12. Statistical Analysis

Graphs and statistical analyses were prepared using GraphPad Prism (V10.6.1). Statistical significance was set at *p* < 0.05. One-way or two-way ANOVA was used to compare more than two groups or to test genotype × treatment effects; Tukey’s test was used for post hoc multiple comparisons.

## 5. Conclusions

This study highlights two key points. First, T-DNA alleles in compact genomic regions can produce complex regulatory outcomes that simple insertion-site annotations fail to capture. In *rtnlb16-1*, the relevant phenotype was identified only after resolving the precise insertion structure, testing the neighboring *MSD1* locus, examining RTNLB16 splice variants, and removing the independent insertion on chromosome 4. This underscores the importance of molecular validation when interpreting T-DNA resources, especially for genes with complex splicing patterns or alternative promoters.

Second, the findings suggest that the physiological functions of plant RTNLB proteins may depend not only on the presence or absence of genes but also on the relative abundance of splice isoforms. Many *Arabidopsis* RTNLB genes encode multiple predicted splice variants, yet most functional analyses treat each locus as a single gene. RTNLB16 exemplifies a case in which isoform stoichiometry appears central to growth, chloroplast performance, hormone responsiveness, and stress tolerance. Future work should determine whether RTNLB16 isoforms form mixed complexes, whether isoform 7 has a distinct localization or regulatory function, and whether altered RTNLB16 ratios change ER tubule geometry or plasmodesmatal aperture.

Overall, our results position RTNLB16 as a dosage-sensitive regulator that links ER membrane architecture to photoperiod-dependent growth and stress physiology. By linking splice-variant balance to ABA responsiveness, dark-induced senescence, defense-related transcription, and iron/redox-associated transcriptional responses, this work expands the functional scope of plant reticulons and provides a framework for dissecting isoform-specific functions within membrane-shaping protein families.

## Data Availability

The RNA-seq dataset analyzed in this study will be available on 1 September 2026, at the NCBI Gene Expression Omnibus under accession number GSE242257. All other data generated or analyzed in this study are included in this article and its Supplementary Information.

## Supplementary Materials

The following supporting information can be downloaded at: https://www.mdpi.com/article/10.3390/plants15132022/s1, Figure S1: MSD1 activity and protein expression in Salk_122275 homozygous plants.; Figure S2: Genomic sequence viewer of AT3G10915 and AT3G10920, indicating the correct and exact localization of the T-DNA inserts in Salk_122275 and Salk_020022; Figure S3: *rtnlb16-2* has a wild-type-like appearance; Figure S4: Effect of short-term transition from CLL to LD on *rtnlb16-1* plants; Figure S5: Photoperiod-dependent growth phenotype in mature flowering *rtnlb16-1*; Figure S6: Co-localization of RTNLB16.5:GFP and the ER-mCherry marker in the *N. benthamiana* epidermis; Figure S7: Overexpression of RTNLB16 does not impact growth; Figure S8: CRISPR-Cas9 targeting of RTNLB16 does not cause a visible growth phenotype; Table S1: Primers list; Table S2: GO biological Processes enrichment for CLL-exclusive DEGs; Table S3: GO biological Processes enrichment for LD and CLL-common DEGs; Table S4: GO biological Processes enrichment for LD-exclusive DEGs; Table S5: GO Biological Processes enrichment for 509 DEGs in Figure 7C; Table S6: Differential expression of iron homeostasis genes.
