# Supplementary material for: A Splice-Variant Imbalance of Reticulon-like Protein 16 (RTNLB16) Disrupts Growth and Decreases Sensitivity to ABA and Dark-Induced Senescence in Arabidopsis
Source: Plants (Basel). 2026 Jun 30;15(13):2022. doi: 10.3390/plants15132022 (PMC13364490; doi:10.3390/plants15132022)
Supplement: Supplementary file 1 [file plants-15-02022-s001.zip › Supplementaty Figures RTLNB16 paper.pdf]

Supplementary Figures.

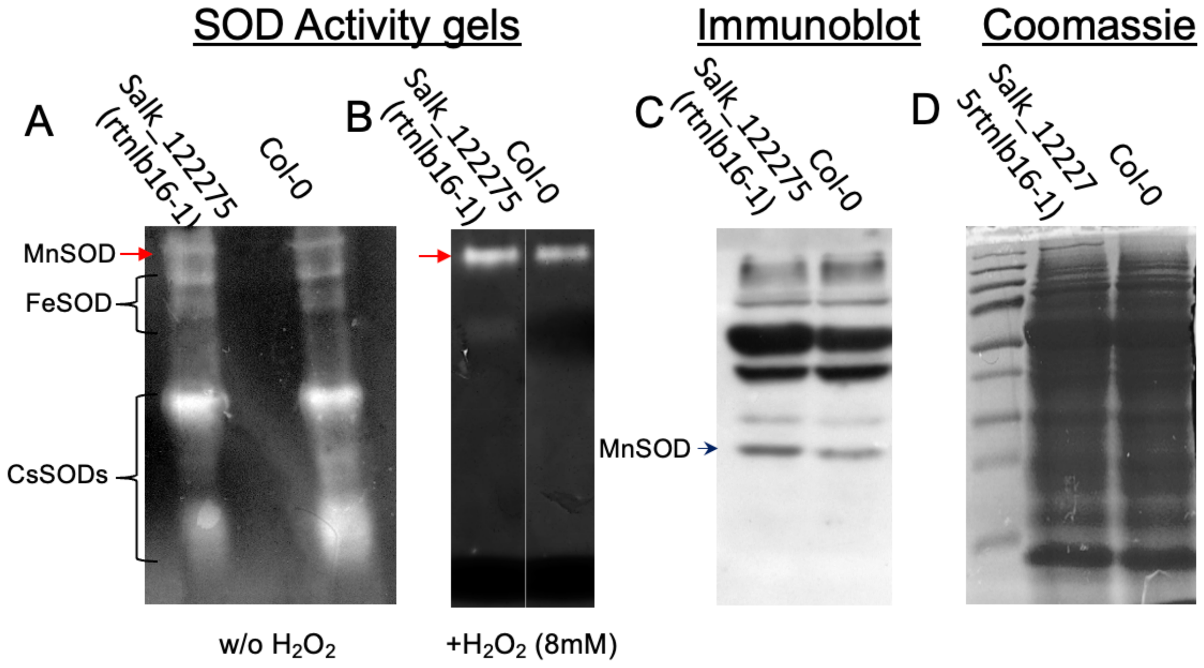

Figure S1. MSD1 protein abundance and MnSOD activity in Salk_122275/*rtnlb16-1*.
(**A**) Native SOD activity gel of leaf extracts from Salk_122275/*rtnlb16-1* and Col-0 without H_2O_2 treatment, showing the positions of mitochondrial MnSOD, FeSOD, and cytosolic SOD activity bands. (**B**) Native SOD activity after treatment with 8 mM H_2O_2, which inhibits H_2O_2-sensitive SOD isoforms and allows visualization of H_2O_2-resistant mitochondrial MnSOD/MSD1 activity. (**C**) Immunoblot of leaf protein extracts probed with anti-MSD1 antibody. (**D**) Coomassie-stained gel used as a loading control. Comparable MnSOD activity and MSD1 protein abundance in Col-0 and Salk_122275/*rtnlb16-1* indicate that the neighboring *MSD1* locus is not detectably disrupted in this line.

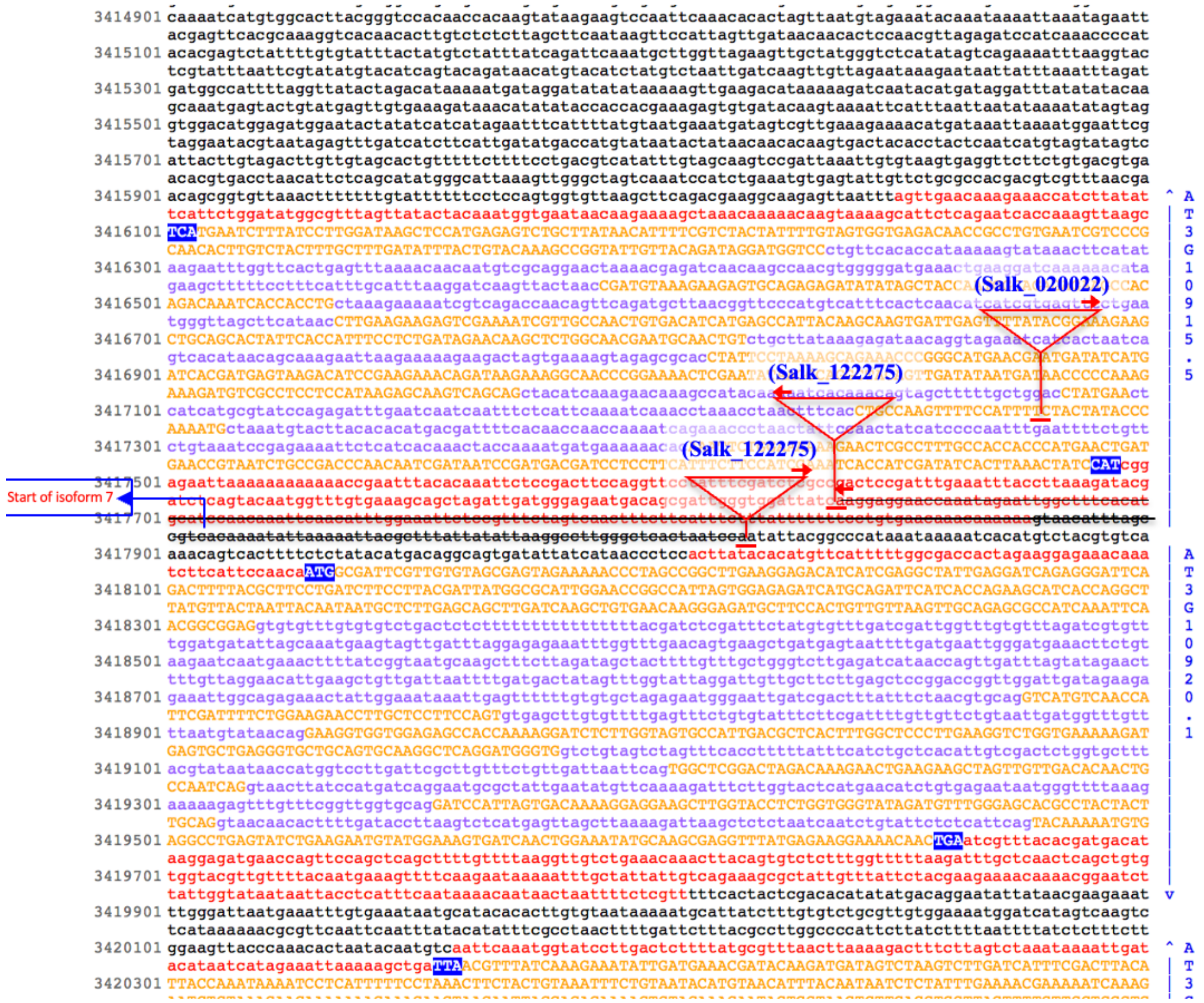

Figure S2. Genomic sequence context of the Salk_122275 and Salk_020022 insertions at the *RTNLB16*/*MSD1* locus. Sequence view of the genomic region containing *RTNLB16* (AT3G10915) and *MSD1* (AT3G10920), showing the experimentally mapped T-DNA insertion positions in Salk_122275/*rtnlb16-1* and Salk_020022/*rtnlb16-2*. The black strike-through indicates the genomic segment deleted during the Salk_122275 insertion event, and the transcription start site of *RTNLB16* isoform 7 is indicated. The *RTNLB16* sequence is shown according to splice variant 5. Uppercase orange letters indicate exons, purple letters indicate introns, red letters indicate UTRs, and black letters indicate intergenic, promoter, or UTR sequences associated with other isoforms.

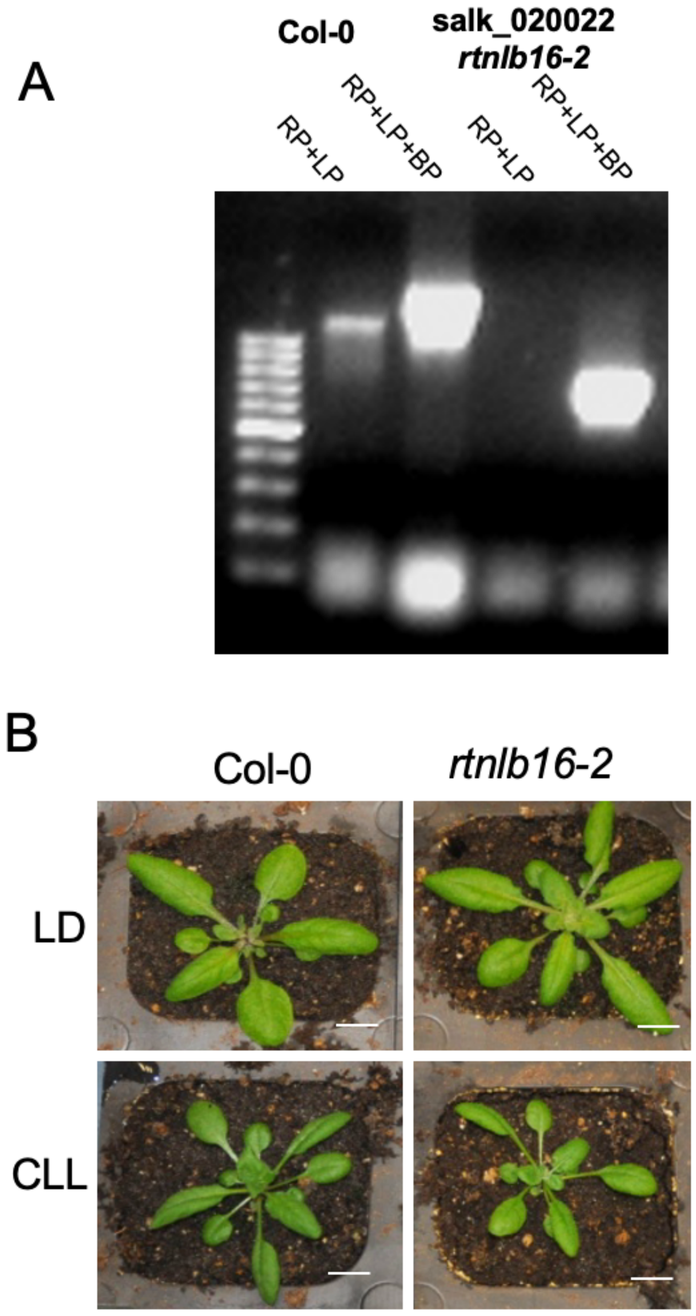

Figure S3. *rtnlb16-2* has a wild-type-like appearance.
(**A**) PCR genotyping of Salk_020022/*rtnlb16-2* using gene-specific primers (RP and LP) and a T-DNA border primer (BP), confirming the insertion allele in the mutant. (**B**) Representative three-week-old Col-0 and *rtnlb16-2* plants grown under LD or CLL conditions. The *rtnlb16-2* line shows no obvious rosette growth or chlorosis phenotype under either light regime. Scale bars are shown in the panels =1cm.

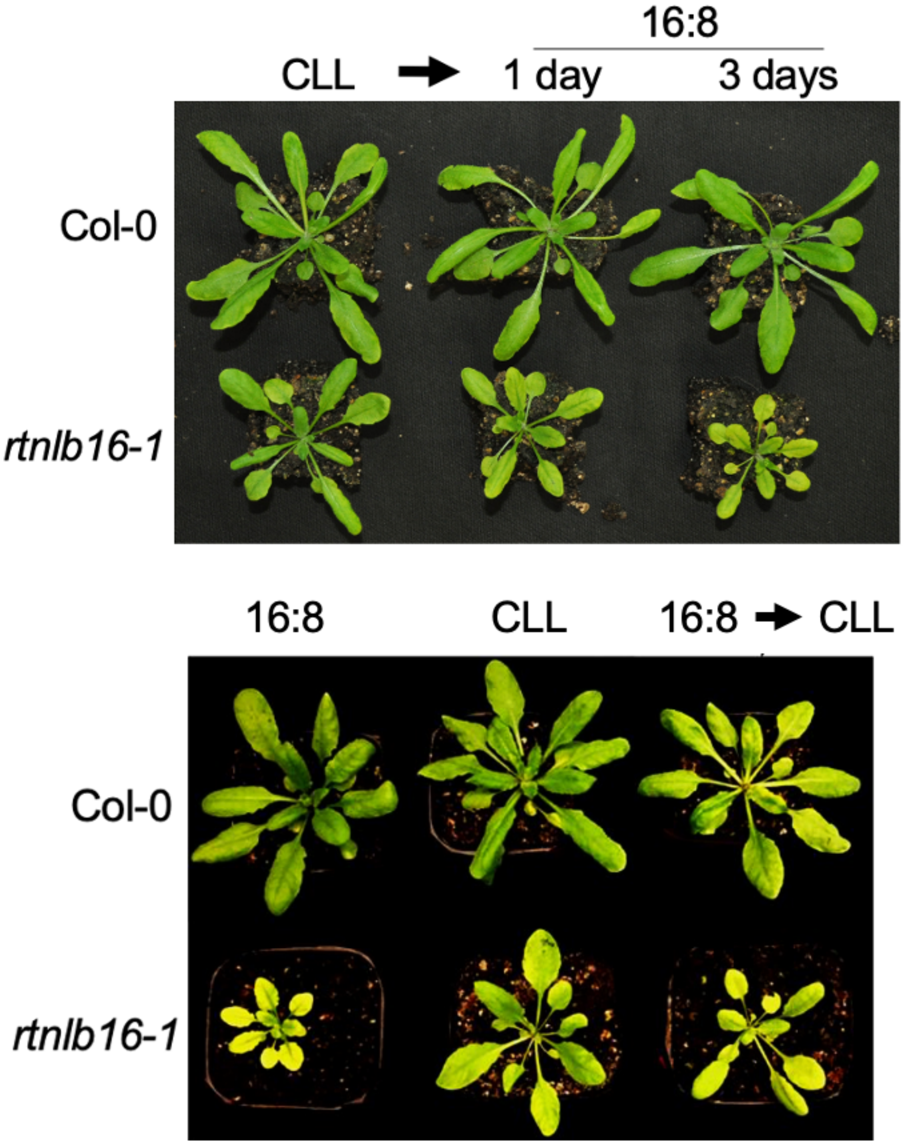

Figure S4. Rapid response of *rtnlb16-1* to transitions between CLL and LD conditions.
Top: Three-week-old Col-0 and *rtnlb16-1* plants grown under CLL and imaged before transfer and one or three days after transfer to LD conditions (16 h light/8 h dark). Bottom: Four-week-old plants initially grown for two weeks under CLL and then maintained under CLL, kept under LD, or returned from LD to CLL for two days. Representative images show rapid worsening of the *rtnlb16-1* phenotype after exposure to LD and partial visual recovery when plants are returned to CLL.

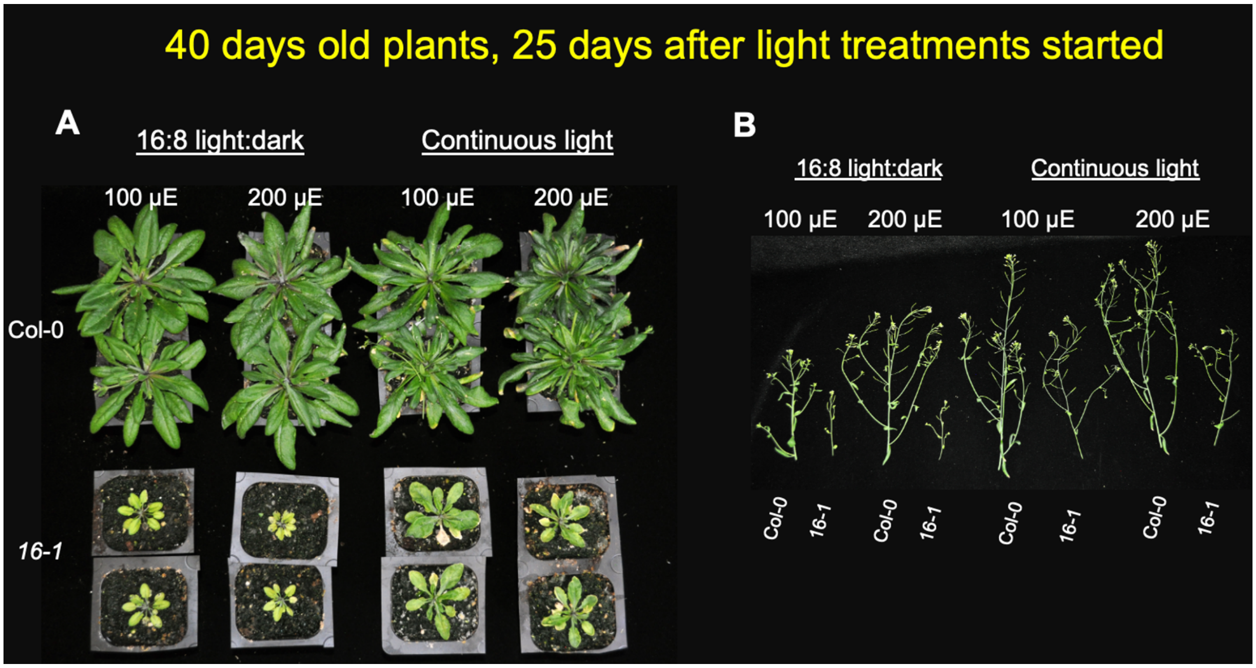

Figure S5. Photoperiod-dependent phenotype of mature flowering rtnlb16-1 plants under different light intensities. (**A**) Rosette phenotypes of 40-day-old Col-0 and rtnlb16-1 plants 25 days after transfer from CLL to either LD (16 h light/8 h dark) or continuous light (CL) at 100 or 200 µmol m^{-2} s^{-1}. (**B**) Inflorescence stems from plants grown under the corresponding light regimes and intensities; stems were excised to allow clear visualization of rosettes in panel A. The images show that the rtnlb16-1 vegetative and reproductive phenotypes are more severe under LD than under CL, even at matched light intensities.

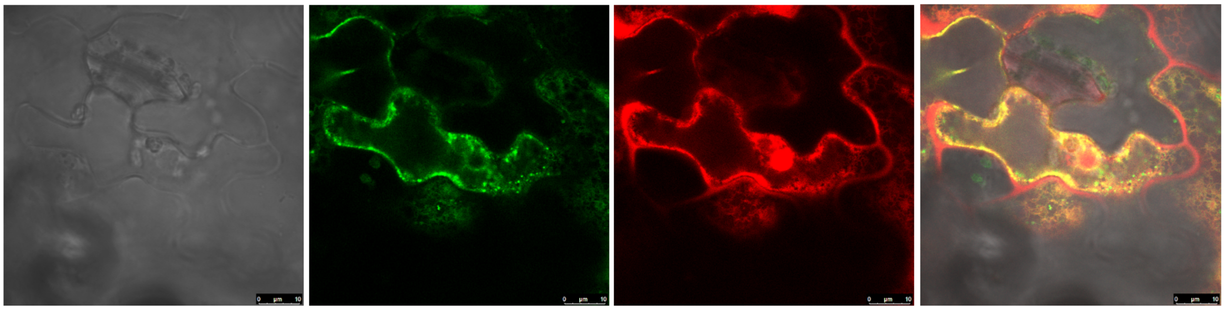

Figure S6. Co-localization of RTNLB16.5-GFP with an ER marker in N. benthamiana epidermal cells. Confocal images of Nicotiana benthamiana leaf epidermal cells transiently co-expressing RTNLB16.5-GFP and ER-rk:mCherry. DIC/brightfield, GFP, mCherry, and merged channels are shown. RTNLB16.5-GFP partially overlaps with the ER marker along cortical ER strands and also localizes to punctate structures. Scale bars are shown in the panels.

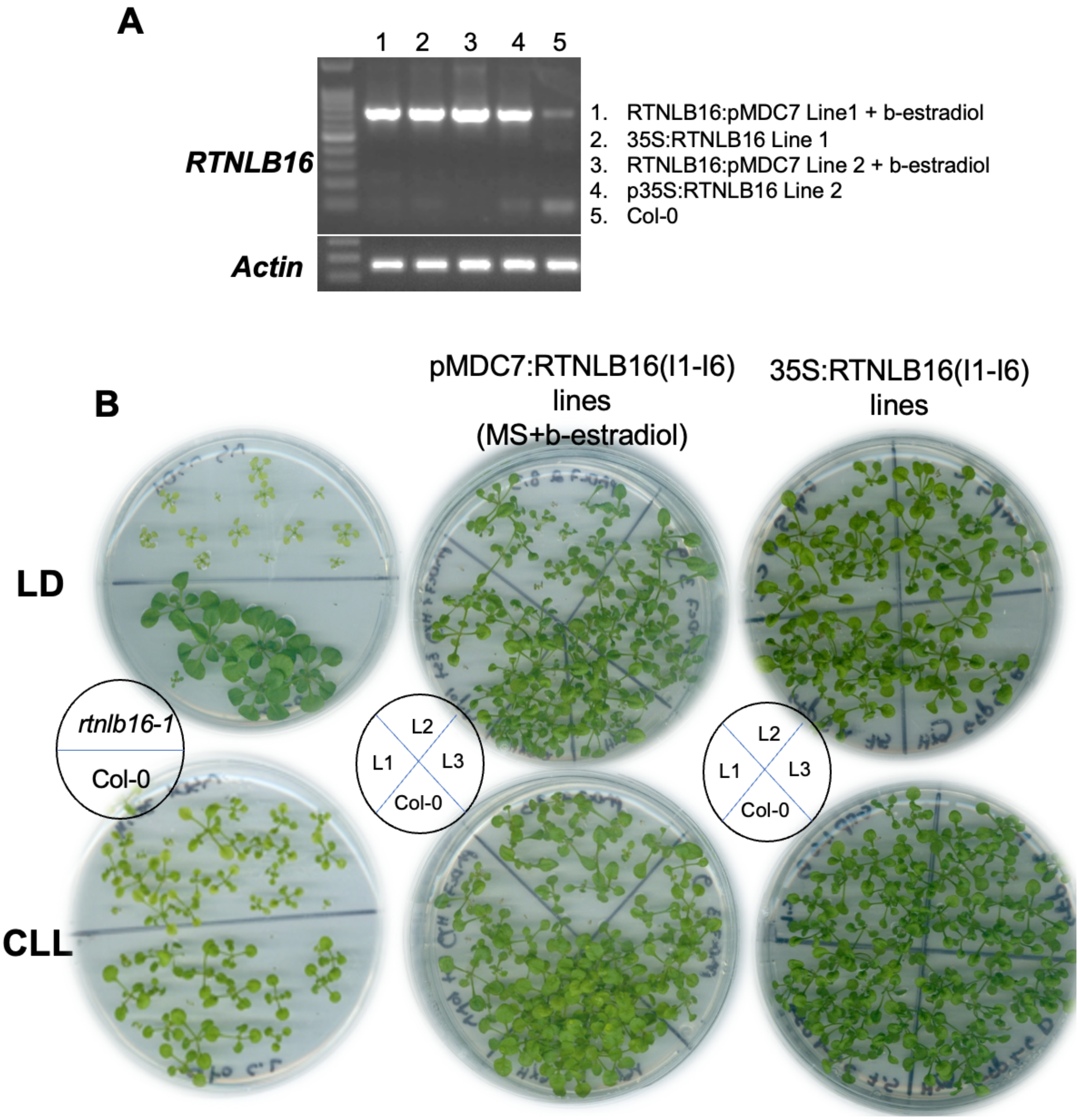

Figure S7. Overexpression of *RTNLB16* isoforms 1–6 does not phenocopy *rtnlb16-1*.
(**A**) RT-PCR/PCR amplification of *RTNLB16* in representative transgenic lines expressing the *RTNLB16* genomic region containing isoforms 1–6 from either a β-estradiol-inducible pMDC7 cassette or the constitutive CaMV35S promoter. *ACTIN* was amplified as a control; lane identities are indicated beside the gel. (**B**) Representative two-week-old transgenic seedlings grown under LD or CLL conditions compared with Col-0 and *rtnlb16-1*. Three independent lines per construct (L1–L3) are shown. Despite elevated *RTNLB16* expression, overexpression of isoforms 1–6 does not reproduce the severe *rtnlb16-1* growth phenotype.

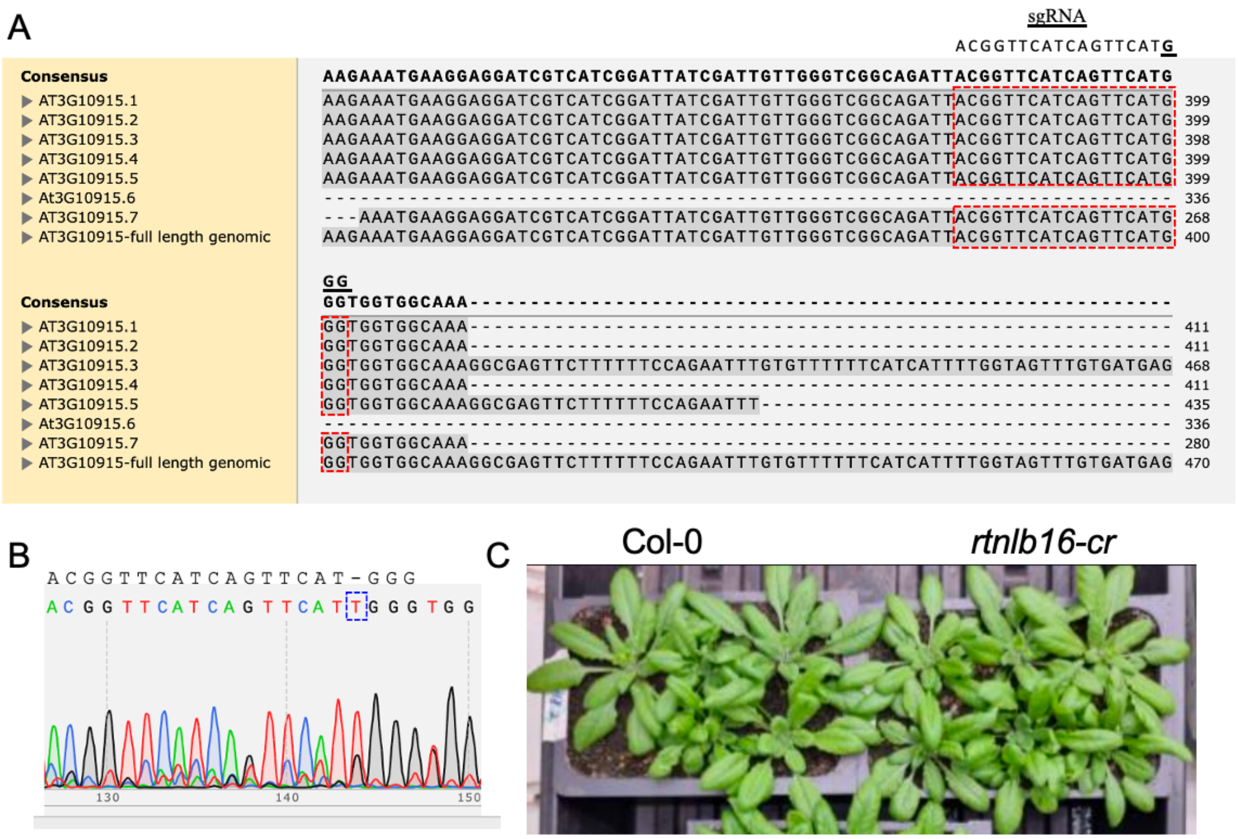

Figure. S8 CRISPR-Cas9 disruption of major *RTNLB16* isoforms does not cause a visible growth phenotype. (**A**) Alignment of *RTNLB16* splice variants showing the sgRNA target site and PAM in a shared coding region targeted by CRISPR-Cas9. The target site is present in the major *RTNLB16* isoforms and is predicted to disrupt isoforms 1–5 and 7 while leaving isoform 6 unaffected. (**B**) Sanger sequencing chromatogram of the *rtnlb16-cr* allele showing a single thymidine insertion near the sgRNA/PAM site, generating a frameshift and premature stop codon in the targeted isoforms. (**C**) Representative Col-0 and *rtnlb16-cr* plants showing no visible rosette growth defect under the conditions shown. This control indicates that loss of the major *RTNLB16* isoforms alone is not sufficient to reproduce the *rtnlb16-1* phenotype.
